# The Physiological Action of Protoxide of Nitrogen

**Published:** 1870-08

**Authors:** John. J. Colton

**Affiliations:** Philadelphia, Pa.


### SELECTED ARTICLES.

### ARTICLE IV.

### *The Physiological Action of Protoxide of Nitrogen.*

By John. J. Colton, M. D., of Philadelphia, Pa.

It has been a disputed point among scientific men, ever since the protoxide of nitrogen came into general use as an anæsthetic, whether its action depends upon oxidation or whether the carbonic acid generated by the ordinary disintegration of tissue, or, by superoxydation, produces the anæsthesia. Eminent gentlemen in this country and Europe have maintained (and with some plausibility) that carbonic acid, by its action upon the nerve centres, produces the anæsthesia, arriving at this conclusion from the fact that evidences of asphyxia, are in some cases, so manifest, while others have held to the theory of oxidation.

Having administered this gas to many thousands of persons, hundreds of times during as many successive days, and having carefully watched its effects upon the system under the greatest variety of circumstances and conditions, I have maintained that its action must depend upon oxidation and that the indications of asphyxia (slight in ordinary cases) are merely an incidental effect rather than the main cause of the anæsthesia.

The following experiment, it seems to me, clearly establishes this theory.

Exp. 1. Take two jars of equal size filled with lime water, pass a definite amount of air, as it comes from the lungs, through one solution, and it is rendered turbid ; pass an equal amount of nitrous oxide, as it escapes from the lungs, through the other solution, and it is also rendered turbid, but to a greater degree than the first, indicating the presence of carbonic acid in greater quantity.

Exp. 2 Breathe through a tube into a solution of litmus blue, and it is changed to red. It will take, we will say 15 seconds, to effect the change (the time depending upon the strenght and quantity of the solution). Now breathe nitrous oxide gas through another solution of the same strength and quantity, and we shall notice the change of color in from 10 to 12 seconds, indicating the elimination of carbonic acid to a greater extent than the normal amount, while breathing the gas, as in the first experiment.

Exp. 3. Take a full inspiration of air, retain it in the lungs one minute, and there are no indications of anæsthesia. Now try the same experiment with the gas, and the anæsthetic effect is manifest almost to insensibility while there are no indications of asphyxia.

We have thus demonstrated, by the first two experiments, that while breathing protoxide of nitrogen, an increased amount of carbonic acid is exhaled, and as this excess *must* be produced by oxidation, we *reasonably* infer that the action of the protoxide is oxidation ; and the third experiment furnishes corroborative testimony, though of a negative character; for if the retention of carbonic acid cause the anæsthesia why are there not some indications when air is retained in the lungs ?

If we inhale pure nitrogen, we still exhale carbonic acid for a time, owing to the union of the oxygen previously introduced into the system, with the carbon and hydrogen of the tissues, but the amount is sensibly diminished. Now, this diminished amount of carbonic acid might have been anticipated, since we have not furnished sufficient oxygen to produce the normal amount. But on the other hand, when we find an increased amount of carbonic acid we infer a corresponding increase of oxidation.

But it is objected that nitrous oxide is not a mixture of gases like the air, but a chemical compound, and therefore it does not act by oxidation. Let us see. Exp. 4 Plunge a lighted taper into the gas, and it burns with greatly increased brilliancy ; the heated elements of the taper presenting a stronger affinity for the oxygen than the nitrogen does, take it and form the new combinations, carbonic acid and water. This fact shows the affinity of the elements composing nitrous oxide to be feeble.

Reasoning, now, from analogy, we might be led to infer that similar results would follow the introduction of the gas into the system, and such we find to be the case. The oxygen of the gas, by its stronger affinity, seizes upon the carbon and hydrogen of the tissues, with the same results of combination as in the case of the taper; in other words, we have superoxidation.

But it is objected that *pure oxygen* exerts a less powerful action upon the system than the gas which is two thirds nitrogen. The answer is simply, that the blood absorbs but a small percentage of oxygen, so that when it is introduced into the lungs it finds its way into the system slowly, while nitrous oxide is rapidly absorbed and conveyed to the tissues to be given up for combination.

Exp 5. If one takes a full inhalation of the gas, he instantly feels a thrill throughout the entire system. This is the incipient stage of anæsthesia, and the rapidity of its action is another proof of the theory of oxidation, for if these sensations were caused by the accumulation of carbonic acid, we should hardly anticipate such a result in the course of a few seconds.

It is still further objected that the asphyxiated appearance of persons under its influence, indicating a superabundance of carbonic acid in the system, suggests, at least, that it is the cause of the anæsthesia. The third, fourth, and fifth experiments furnish proof of the absurdity of this conclusion.

Moreover, the principle evidence of asphyxia ordinarily manifested is the cyanosed tint of the lips, which is in part, produced by pressure in holding them tightly upon the inhaling tube, while in certain cases, in which these evidences are more striking, there is a cessatiou of breathing caused by the tongue falling back upon the epiglottis; by removing this obstruction, and allowing the patient a breath of air, the symptoms of asphyxia disappear. If the inhalation be prolonged to extreme limits, we shall also notice the asphyxiated appearance owing to the accumnlation of carbonic acid.

The above indications furnish the main argnment in favor of the carbonic acid theory. That there accumulates in the system a larger than the normal amount of carbonic acid is evident, and this is entirely consistent with the theory of oxidation, but this accumulation is not in sufficient amount in ordinary cases to attract attention, certainly not enough to produce profound insensibility. Moreover, the effects of nitrous oxide upon the system are not such as we should anticipate, were they caused by the action of carbonic acid, for it is depressing in its influence. Even if a slight impediment is offered to its elimination from the lungs, " a feeling of discomfort and depression increasiug with the duration of the interruption is speedily felt."

Now, while inhaling the nitrous oxide, although there is an increase of carbonic acid in the system, yet, if it be in sufficient amount to produce anæsthesia, how shall we explain the immediate reaction, and the agreeable sensations while inhaling, as well as for some time subsequent.

In the ordinary use of stimulants, there follows " a period of depression corresponding to the exaltation of the functions excited," yet persons who have submitted to the influence of nitrous oxide give no indications of this character. On the contrary, there ordinarily follows a period of exhilaration, just what might be anticipated from an excess of oxygen.

Tne system resumes its normal conditions as soon as the oxygen has been disposed of by union and the carbonic acid and other products of oxidation eliminated, which process is very rapid—while chloroform, ether, alcohol, and other narcotics and stimulants linger in the system much longer, and by their prolonged action upon the nerve centres, produce their depressing effects and obstruct oxidation.

But the wonderful power of the nitrous oxide yet remains to be explained.

It is a well-known principle of chemistry, that elements in the nascent state, just liberated from union, exhibit remarkable characteristics and properties.

Nitrogen and hydrogen, for example, placed in a jar together, manifest no affinity for each other, and cannot be induced to unite even by the application of the most intense heat—but let them come together just at the moment of liberation from other compounds, and they combine with the utmost avidity. This is the precise condition in which the oxygen of the protoxide is found, as it enters into combination with the tissues of the body—just liberated from its union with nitrogen, it is in its nascent state—the active condition, and thus it exhibits its extraordinary powers of oxidation.

I have in this brief and cursory manner endeavored to prove, (1) that persons breathing protoxide of nitrogen exhale a larger amount of carbonic than while breathing common air: and (2) that its action is essentially oxidation, and that the excess of carbonic acid in the system is merely incidental, and in nowise sufficient to produce anæsthesia; and, (3) that its inoffensive effects are due to its action as an oxydizer; and (4) that no subsequent depression follows but rather a rapid reaction; because, acting by oxidation, the products of its combinations are speedily eliminated from the system; and, (5) that its remarkably powerful action is due to its entering into new combinations, just at the moment of its liberation, when in its nascent state.

I shall reserve for a future article what I had intended to say of its uses and abuses, and close by expressing my profound conviction that the pure protoxide of nitrogen, administered within proper limits, is harmless in its action, while if made from contaminated materials; if at too high a temperature; if used immediately after it is made; if the impurities ordinarily found in it be not removed; if inhaled in sufficient quantity to produce profound anæsthesia, after being prepared for a few days, or if the administration of the *pure gas* be persisted in beyond *certain limits* it is capable of an immense amount of mischief. At the same time I would, express the earnest desire that a free and full discussion of the subject may afford a better knowledge of its physiological action and increase its value as an agent for the relief of human suffering.—*Medical and Surgical Reporter.*

